# Morphology, Biophysical Properties and Protein-Mediated Fusion of Archaeosomes

**DOI:** 10.1371/journal.pone.0039401

**Published:** 2012-07-06

**Authors:** Vid Šuštar, Jasna Zelko, Patrizia Lopalco, Simona Lobasso, Ajda Ota, Nataša Poklar Ulrih, Angela Corcelli, Veronika Kralj-Iglič

**Affiliations:** 1 Laboratory of Clinical Biophysics, Chair of Orthopaedics, Faculty of Medicine, University of Ljubljana, Ljubljana, Slovenia; 2 Department of Medical Biochemistry, Biology and Physics, University of Bari Aldo Moro, Bari, Italy; 3 Department of Food Science and Technology, Biotechnical Faculty, University of Ljubljana, Ljubljana, Slovenia; 4 IPCF-CNR, Bari, Italy; 5 Biomedical Research Group, Faculty of Health Sciences, University of Ljubljana, Ljubljana, Slovenia; Aristotle University of Thessaloniki, Greece

## Abstract

As variance from standard phospholipids of eubacteria and eukaryotes, archaebacterial diether phospholipids contain branched alcohol chains (phytanol) linked to glycerol exclusively with ether bonds**.** Giant vesicles (GVs) constituted of different species of archaebacterial diether phospholipids and glycolipids (archaeosomes) were prepared by electroformation and observed under a phase contrast and/or fluorescence microscope. Archaebacterial lipids and different mixtures of archaebacterial and standard lipids formed GVs which were analysed for size, yield and ability to adhere to each other due to the mediating effects of certain plasma proteins. GVs constituted of different proportions of archaeal or standard phosphatidylcholine were compared. In nonarchaebacterial GVs (in form of multilamellar lipid vesicles, MLVs) the main transition was detected at T_m_ = 34. 2°C with an enthalpy of Δ*H* = 0.68 kcal/mol, whereas in archaebacterial GVs (MLVs) we did not observe the main phase transition in the range between 10 and 70°C. GVs constituted of archaebacterial lipids were subject to attractive interaction mediated by beta 2 glycoprotein I and by heparin. The adhesion constant of beta 2 glycoprotein I – mediated adhesion determined from adhesion angle between adhered GVs was in the range of 10^−8^ J/m^2^. In the course of protein mediated adhesion, lateral segregation of the membrane components and presence of thin tubular membranous structures were observed. The ability of archaebacterial diether lipids to combine with standard lipids in bilayers and their compatibility with adhesion-mediating molecules offer further evidence that archaebacterial lipids are appropriate for the design of drug carriers.

## Introduction

**Figure 1:**
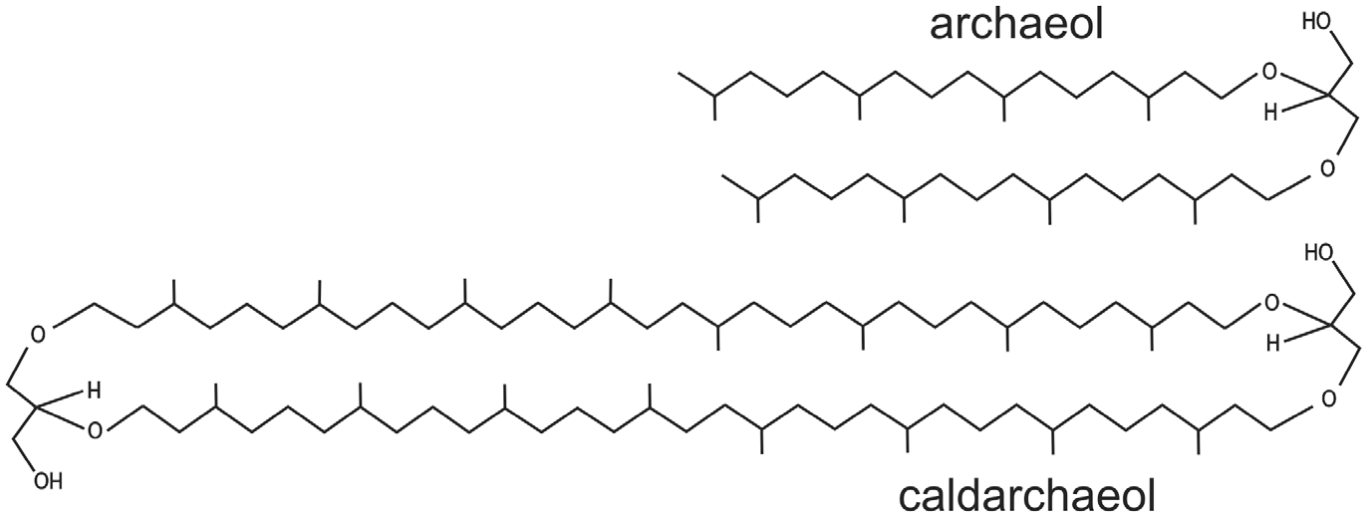
Basic archaeal lipid constituents. Archaeol and caldarchaeaol.

The archaebacterial phospholipids and glycolipids are structurally different from those of bacterial and eukaryotic membranes, being diphytanyl glycerol diether compounds [1]–[4] in which isopranoid chain alcohols are linked to glycerol by ether bonds. In some archaeal microorganisms the phytanyl chains can combine and form biphytanyl chains which link to two glycerols (or to one glycerol and one nonitol) forming tetraether bipolar (bolaform amphiphilic) lipids. While diether lipids assemble in bilayers, tetraether bipolar lipids constitute monolayer membranes. Figure 1 illustrates two basic types of archaeal lipids: diphytanyl glycerol or archaeol and so called caldarchaeol. The ether bond and the almost complete absence of unsaturation in the hydrophobic tail of lipids of archaebacteria are considered adaptive traits of microorganisms which are able to thrive in harsh or extreme environments, such as saturated salt solutions [5], anoxic [5] or highly oxidized [6] conditions and hot waters [5], [7]. A different set of life-fundamental enzymes are involved in archaebacterial lipid biosynthesis [8]. Also, different chirality of the glycerol phosphate moiety [3] protects them against hydrolysis by phospholipases secreted by other organisms [9]. These properties could be of advantage in using archaebacterial lipids in human and veterinary medicine and are therefore a subject of increasing interest.

The role of archaebacterial lipids as vaccine adjuvants [10]–[12] and the possibility to use liposomes constituted of archaebacterial lipids as delivery system of drugs, genes and proteins [7], [13] provides an incentive to study interactions between archaebacterial and eukaryotic lipids, as well as to study the effect of different biologically important molecules on the mediated interactions between membranes composed of different lipid species.

Liposomes prepared from the lipid extracts of archaebacteria (archaeosomes), constituted of mixtures of various polar lipids, have been used in reconstitution studies [14], [15], in the study of the characteristics of membrane permeability [16] and as a delivery system in the immune response to specific antigens [10], [13], [17]. It has been shown that archaeosomes can endure extreme temperatures [18], [19] and resist extreme alkaline-baso-acidic and non-archeal enzyme degradation [19].

Giant lipid vesicles with the dimensions of living cells (GVs) are appropriate for study of properties and interactions of lipids. The advantage of using GVs is that they are large enough to be observed in real time under the phase contrast microscope or fluorescent optical microscope. GVs made of standard lipids of bacterial and eukaryotic membranes (also named in the following as non-archaebacterial lipids) were thoroughly studied experimentally [20]–[29] and theoretically [30]. In studying complex interactions between membranes constituted of standard phospholipids and proteins, it was found that certain plasma proteins mediate attractive interaction between membranes thereby causing close contact between GVs [31], [32], which is an essential step in some biologically important processes such as fusion and fission of vesicles with the mother membrane.

**Figure 2:**
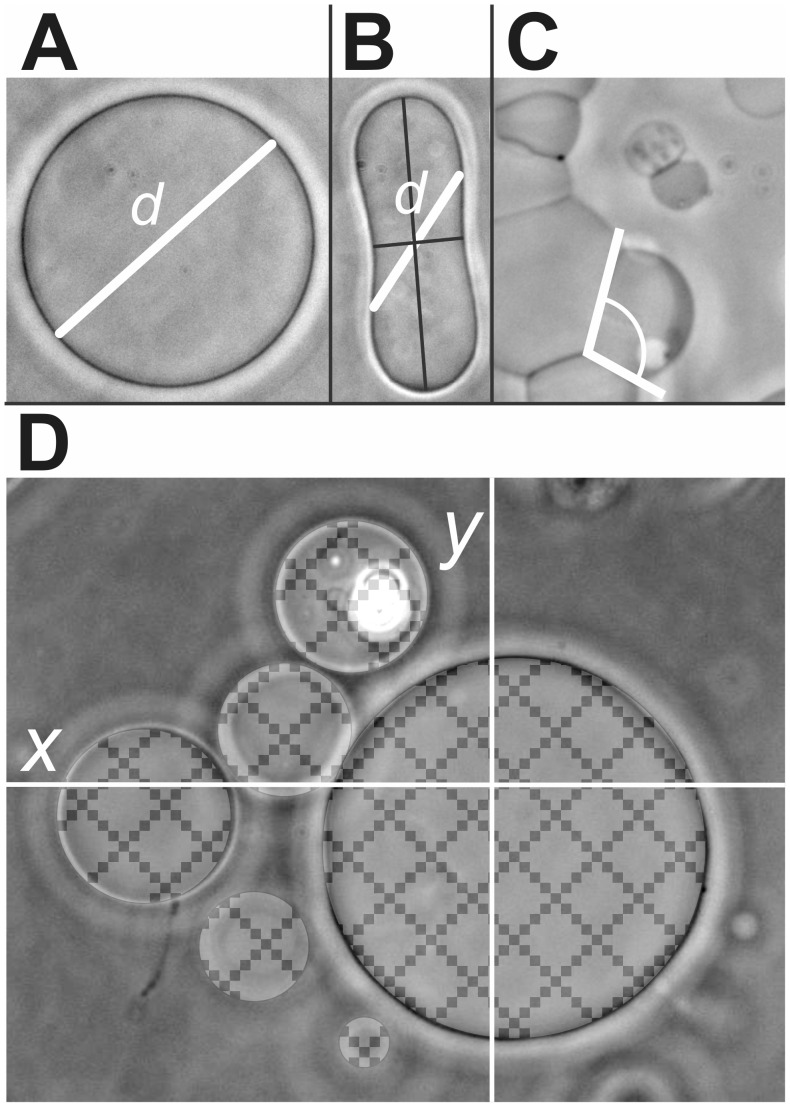
Structural characterisation of archaeosomes. Measurement of GVs’ size (A,B), effective angle of contact between adhered GVs (C) and GVs’ yield (D).

Until now studies involving GVs composed of archaebacterial lipids have mainly considered tetraether bipolar lipids in monolayers. Bagatolli et al. [25] have reported that GVs composed of archaeal tetraether lipids can be formed by the electroformation method, while Cavagnetto et al. [33] studied GVs composed of lipid fractions extracted from the thermophilic archaeobacterium *Sulfolobus solfataricus* mixed with eukaryotic lipids.

It is of interest to further study GVs composed of archaebacterial lipids and their interactions with the molecules in the surrounding solution. In the present work we have studied the GVs constituted of bilayer-forming archaeal diether phospholipids and glycolipids extracted from halophilic microorganisms inhabiting hypersaline environments, such as coastal salterns and continental salt lakes. We describe the shape and size of GVs constituted of different pure archaebacterial diether phospholipids and glycolipids and of mixtures of archaebacterial and non-archaebacterial lipids, by changing the proportions of membrane constituents. Besides assessing the population of GVs for average size and yield, GVs were used to study the mediating effect of two biologically important molecule species that act as anticoagulants and were previously studied in non-archaebacterial GV systems. These molecules are beta 2 glycoprotein I (β2-GPI), which is commonly found in the pheripheral blood of vertebrates and acts as a cofactor in binding certain antibodies to negatively charged lipids [34], [35], and heparin which is also known for its anti-tumour progression effect [36], [37].

**Figure 3:**
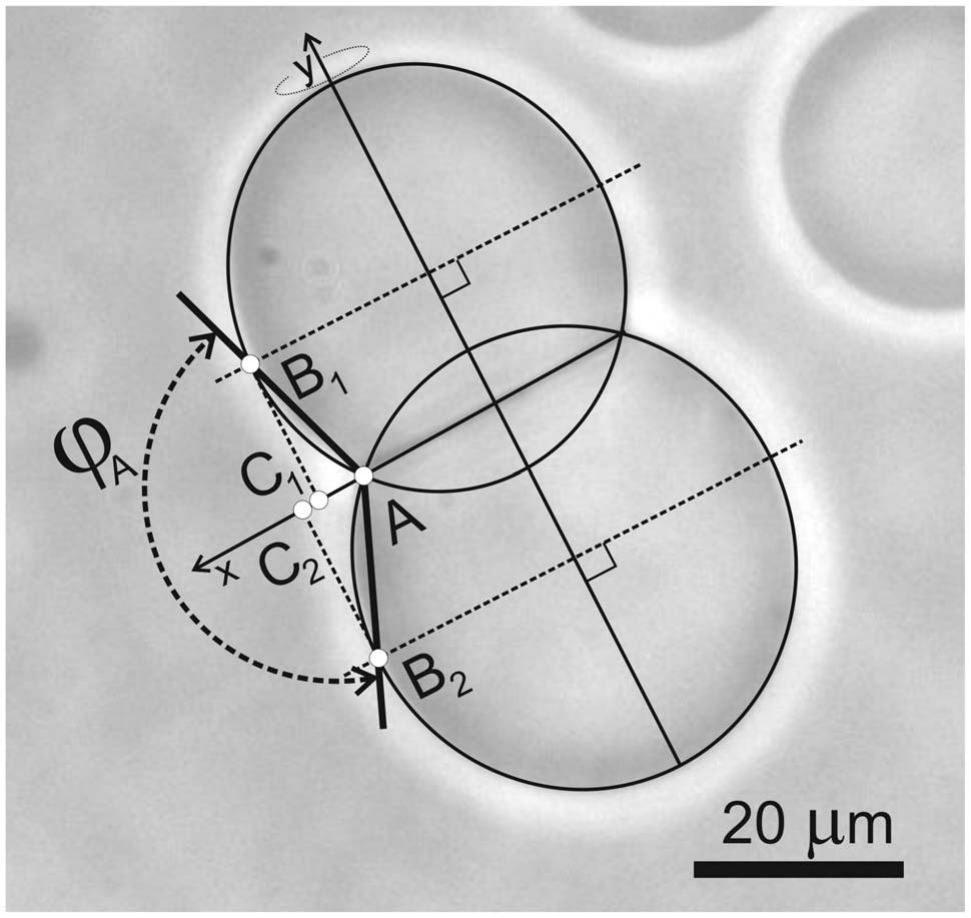
Determination of the adhesion angle. Geometrical parameters of adhered GVs which are needed to assess the adhesion constant as described in the text.

## Materials and Methods

### Chemicals

Synthetic lipids 1-palmitoyl- 2-oleoyl-sn-glycero-3-phosphocholine (POPC, catalogue number 850457), 1,2-dipalmitoyl-sn-glycero-3-phosphocholine (DPPC, 850355), plant cholesterol (Ch, 700100), cardiolipin sodium salt (710335), phosphatidyl serine (PS, 840034) and archaeal phosphatidyl choline (aPC, 4ME 16∶0 Diether PC 1,2-di-O-phytanyl-*sn*-glycero-3-phosphocholine) (999984) were from Avanti Polar Lipids, Inc., Alabaster, AL, USA.

Archeabacterial lipids, sulfated diglycosyl diphytanylglycerol diether (S-DGD-5), phosphatidylglycerophosphate methyl ester (PGPMe), bisphosphatidylglycerol (BPG) and phosphatidylglycerol (PG) were isolated and purified from cultures of the extreme halophilic archaebacteria *Hbt salinarum* and *Halorubrum* sp MdS1 as previously described [4]. β2-GPI was from Hyphen BioMed, Andresy, France, low molecular weight heparin nadroparin calcium (Fraxiparine Forte, 19.000 UI AXa/ml) was from GlaxoSmithKline, London, UK and 10-N-nonyl acridine orange (NAO, A7847) was from Sigma-Aldrich, St. Louis, MO, USA.

**Figure 4:**
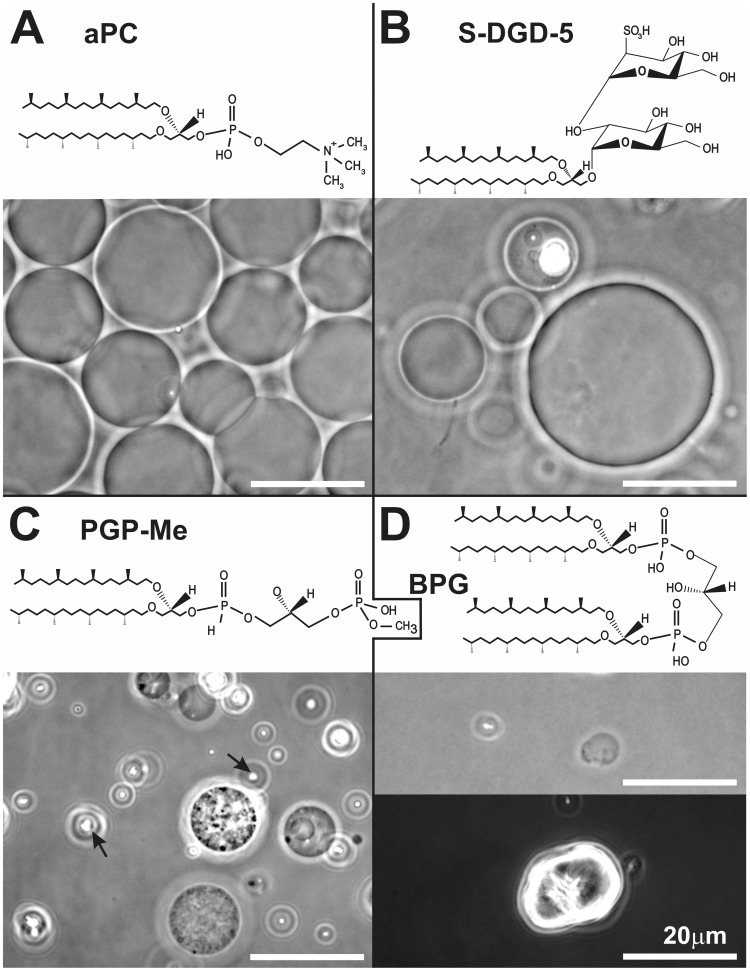
Phase contrast microscope images of archaeosomes composed of different archaebacterial lipids. GVs composed of pure archaebacterial lipids as indicated in individual panels. The arrows in panel C show relatively small crystal-like structures found at the bottom of the chamber. The lower panel D shows a larger crystal-like structure found at the bottom of the observation chamber.

**Figure 5:**
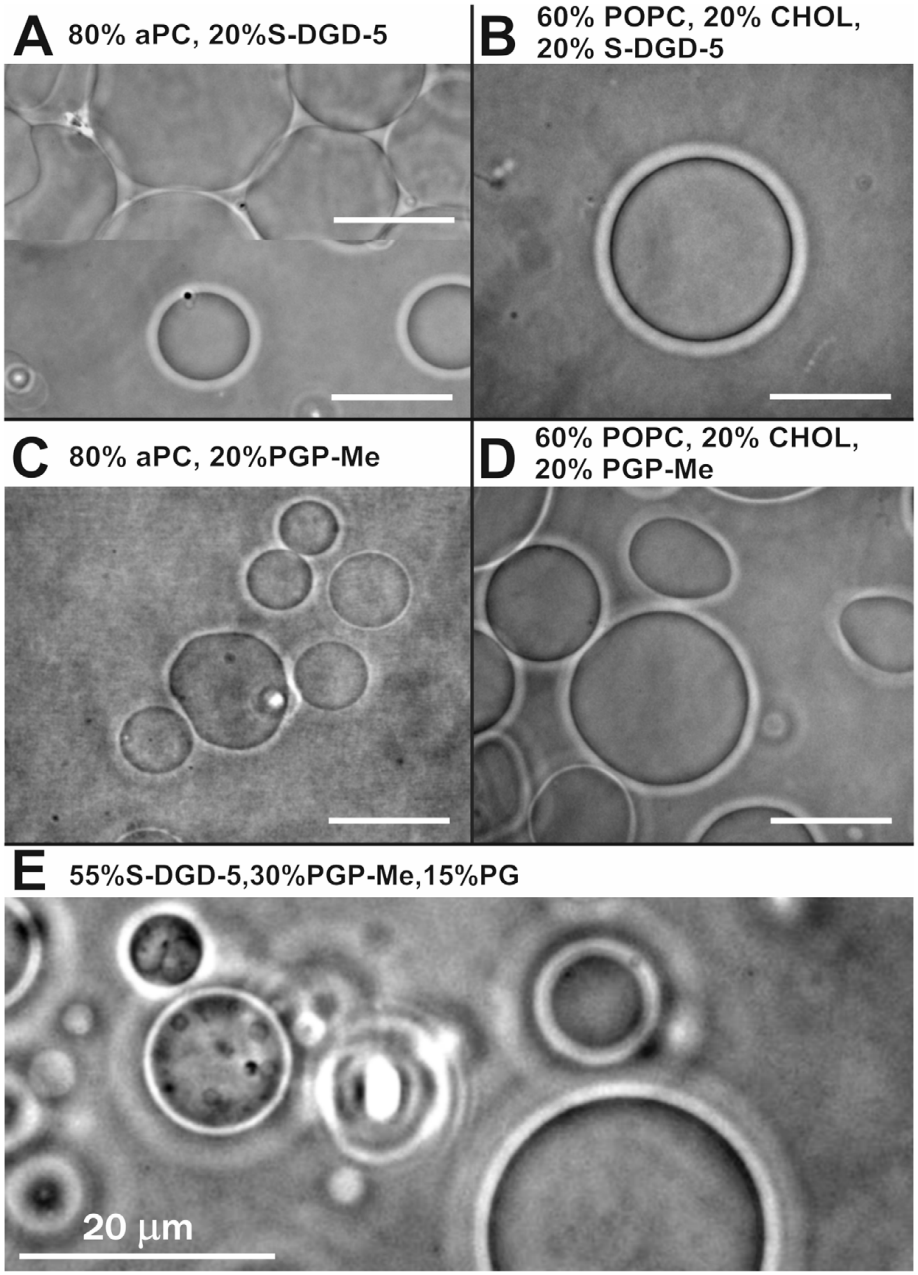
Phase contrast microscope images of archaeosomes composed of different mixtures of archaeabacterial lipids. Archaeosomes composed of different mixtures of archaebacterial lipids as indicated in individual panels. Panel A shows different regions in the same observation chamber.

### Preparation of GVs

Preparation of GVs and experiments were performed at room temperature (23°C). GVs were prepared by the modified electroformation method [38]. Lipids were dissolved in a 2∶1 chloroform/methanol mixture at 1 mg/ml. Lipids were combined in different proportions to examine and compare the effect of lipid composition on the shape of GVs. The exact proportions of lipids are given in Results section. 10 µl of the lipid mixture was applied to each of two platinum rod-shaped electrodes (approximate length 4 cm and diameter 1 mm). The electrodes were left in a low vacuum for 2 h for solvent to evaporate. The lipid-coated electrodes were then placed in a microcentrifuge tube filled with 2 ml of 0.2 M sucrose solution, to constitute an assembled electroformation chamber. An AC electric potential with an amplitude of 5 V and a frequency of 10 Hz was applied to the electrodes for 2 h, which was followed by 2.5 V and 5 Hz for 15 min, 2.5 V and 2.5 Hz for 15 min and finally 1 V and 1 Hz for 15 min. After electroformation, 1800 µl of sucrose solution containing GVs and 3 ml of 0.2 M glucose solution were pipetted into a 5 ml plastic microcentrifuge tube which was sealed with parafilm. Vesicles were left to sediment and stabilise at room temperature for 1 day. For fluorescent staining of GVs, 1 µl of 10-N-nonyl acridine orange (NAO) in 0.5 µM ethanol solution was left to evaporate in an observation chamber shielded from light. 50 µl of GVs in sugar solution was added into the chamber and left for 2–3 minutes shielded from light for NAO to bind to lipids before observation. GVs were created in sucrose and washed by equimolar glucose so that they were heavier than the surrounding solution and sank to the bottom of the observation chamber. This made the observation easier.

**Figure 6:**
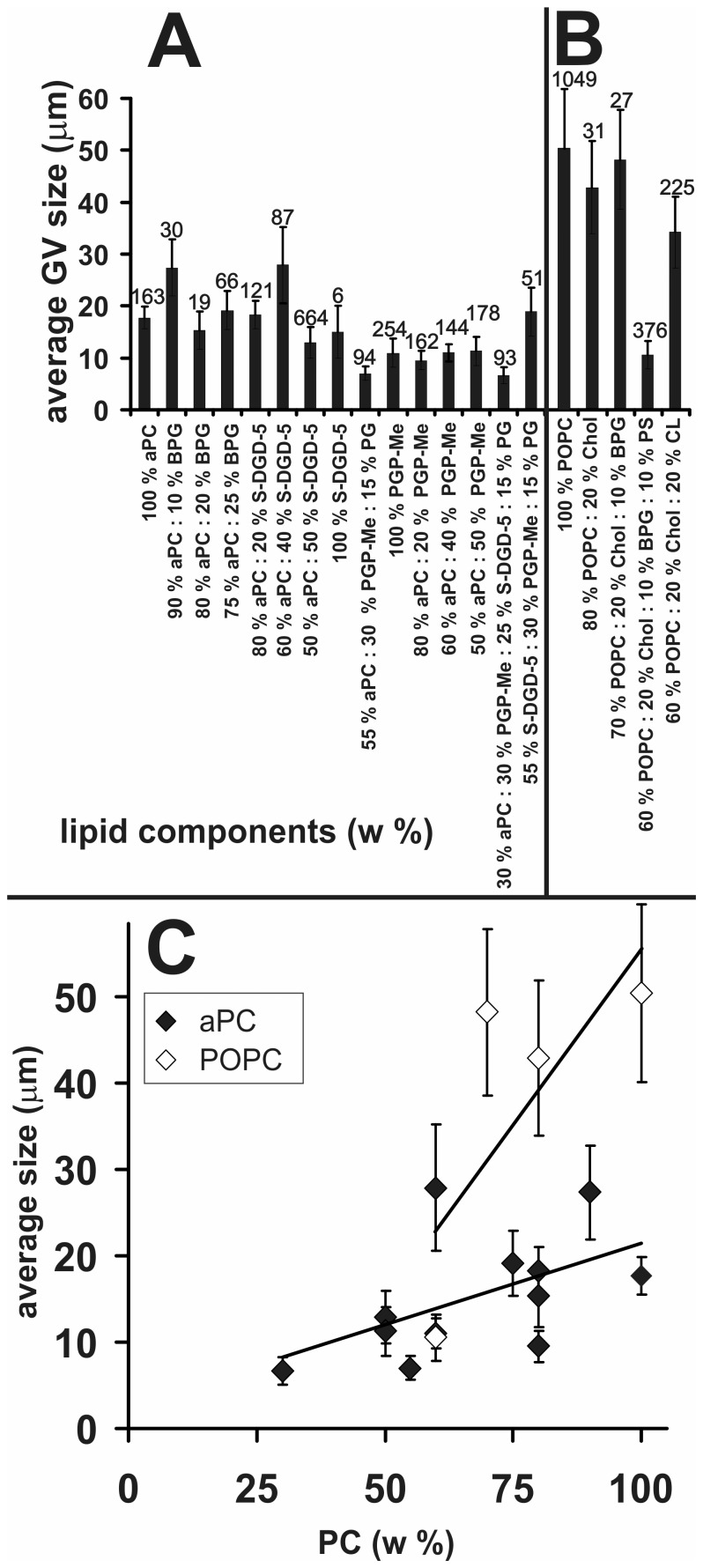
Effect of lipid composition on GVs size. Average size of GVs composed of pure archaebacterial lipids and mixtures of different archaebacterial lipids (A) and of mixtures of non-archaebacterial and archaebacterial lipids (B). Numbers of GVs in each experimet are indicated. Dependence of the average GVs size on the proportion of phosphatidylcholine in the lipid mixture (C). Lines represent best fits of data. Bars represent standard deviations.

**Figure 7:**
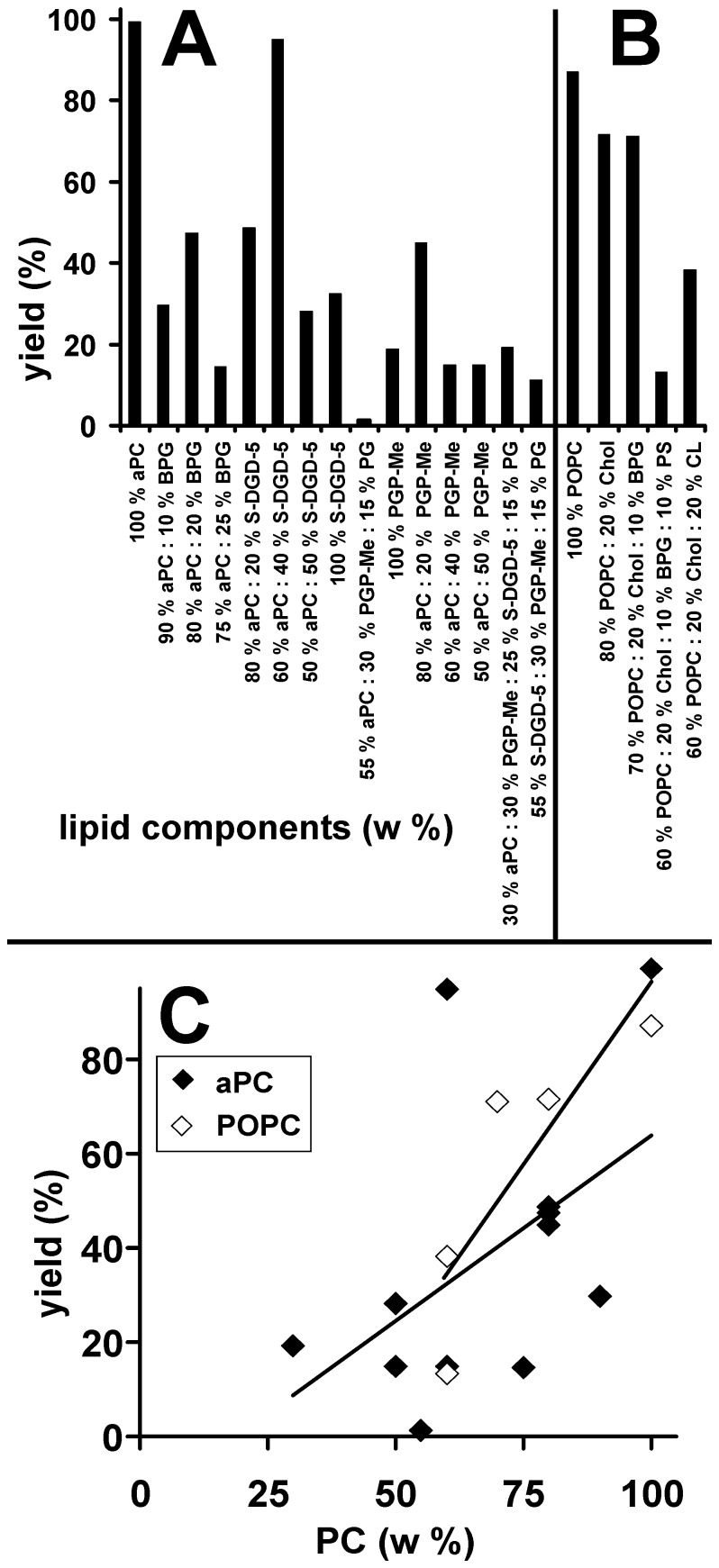
Effect of lipid composition on GVs electroformation yield. Yield of GVs composed of pure archaebacterial lipids and mixtures of different archaebacterial lipids (A) and of mixtures of non-archaebacterial and archaebacterial lipids (B). Dependence of yield on the proportion of phosphatidylcholine in the lipid mixture (C). Lines represent best fits of data.

### Observation of GVs

GV shapes and adhesion between GVs were observed with an inverted microscope (Axiovert 200, Carl Zeiss AG, Oberkochen, Germany). 65 µl of the solution with sedimented vesicles was collected from the bottom of the tube by a pipette and inserted through a circular opening into a 70 µl CoverWell™ Perfusion Chamber (Grace Bio-Labs, Bend, OR, USA). The sample was left for 10 minutes for sedimentation of GVs. After sedimentation, digital micrographs of GVs were taken.

For experiments examining the adhesive effect of the added substance, 5 µl β2-GPI (65, 130, 650, 975 and/or 1820 µg/ml), or 5 µl fraxiparine (diluted in 0.2 M glucose to 50 AXa/ml) were added to the solution of GVs into the perfusion chamber. Images of GVs were taken at different times, starting immediately after addition of fraxiparine to GVs. In the case of β2-GPI concentration-dependent adhesion of GVs, images were taken in the timespan of 10 minutes starting 30 minutes after addition of β2-GPI to GVs. Images were acquired along the horizontal axis of the perfusion chamber.

Observation of GVs labeled with NAO was performed 3–15 minutes after the addition of NAO to the suspension of GVs. The excitation wavelength was 500 nm, while the emission wavelength was 535 nm. The exposure time was 1 second.

### Preparation of Multilamellar Lipid Vesicles (MLVs)

0.5 mg of dried lipid (aPC or DPPC) was dissolved in chloroform/methanol mixture (2∶1, v:v) and transferred into round-bottomed glass flasks. The solvent was evaporated under reduced pressure (17 mbar). 0.2 M glucose:sucrose 5∶3 vol:vol solution was added to dried lipid films to obtain a suspension with final lipid concentration of 0.5 mg/mL. To yield MLVs the suspension was transferred into a glass vial and incubated for 2 hours at 45°C with vortexing every 10 min.

**Figure 8:**
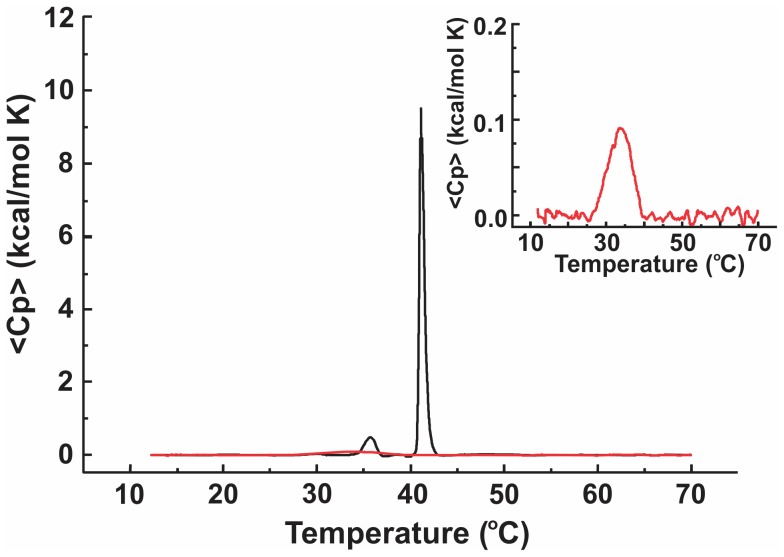
The excess specific heat of MLVs composed of archaebacterial DPPC (aPC) and of non-archebacterial DPPC. Red curve pertains to aPC while black curve pertains to non-archaebacterial DPPC. The inset shows an enhanced view on the peak pertaining to aPC.

### Differential Scanning Calorimetry (DSC)

The phase transition of MLVs prepared from aPC and DPPC lipids in 0.2 M glucose:sucrose 5∶3 vol:vol solution was performed using the Nano DS series III calorimeter (Calorimetry Science, Provo, UT, USA). The sample was transferred into the calorimetric cell and repeatedly heated/cooled in the temperature range from 10°C to 70°C. The heating/cooling rate was 1°C/min. The first DSC scan was used to obtain the phase transition temperature, *T*_m_, the excess specific heat, <*c*_p_> and the enthalpy of the phase transition, Δ*H*_._ Subsequent two scans were used to assess the reversibility of the phase transition. The data were analyzed using the OriginPro software (V. 8.1., OriginLab Corporation, Northampton, USA).

**Figure 9:**
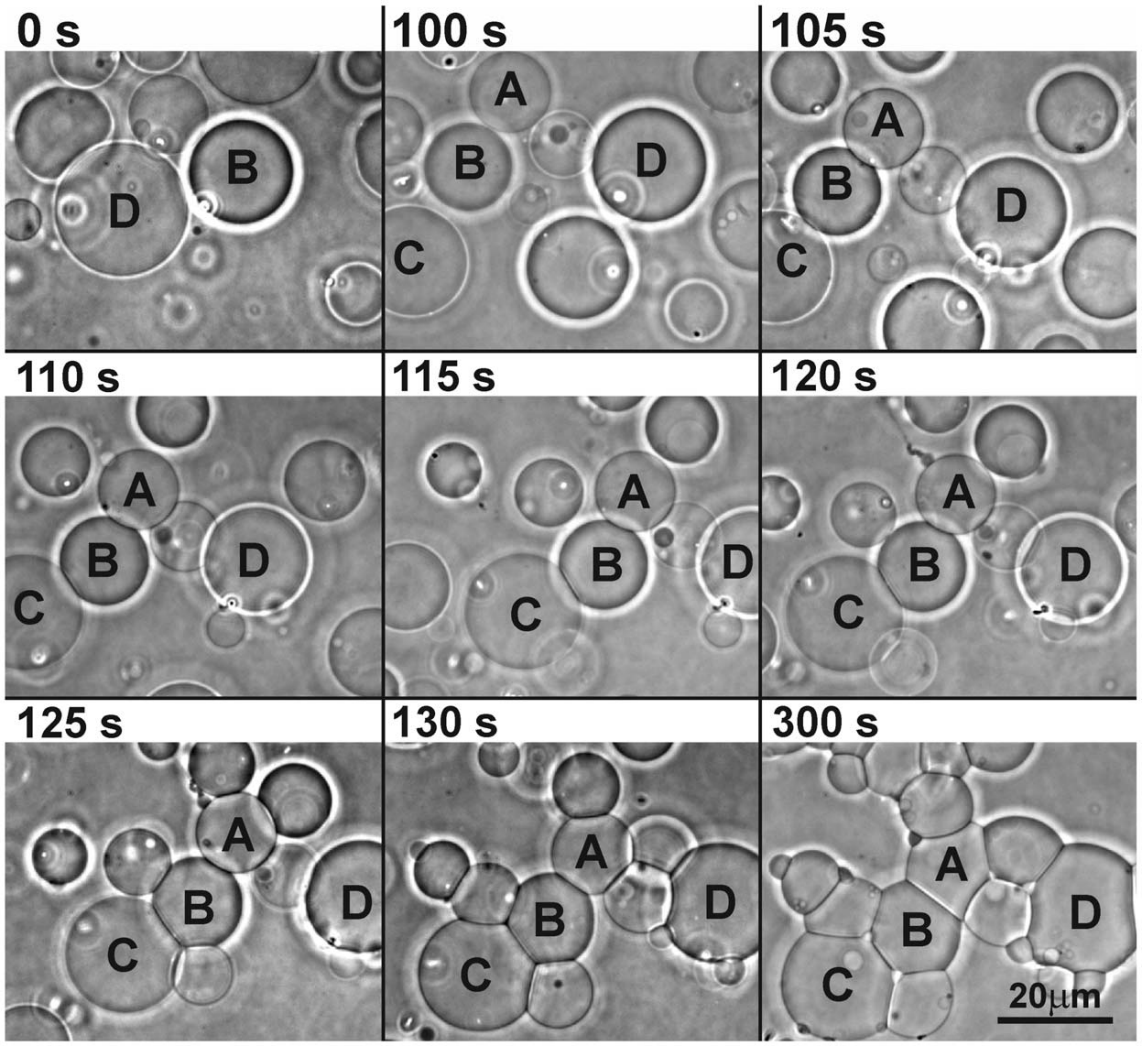
Heparin-induced adhesion of archaebacterial GVs. A sequence of images taken at different times showing gradual adhesion of archaebacterial GVs (composed of 25%BPG and 75% aPC) after the addition of fraxiparine to the suspension of GVs.

### Assessment of Populations of GVs and of Mediated Interaction between Membranes

The average size of GVs within the population was measured by assessment of the dimensions of images recorded using Image J software (V. 1.45 s, NIH, Bethesda, MD, USA). All GVs in the chosen frame were assessed for size. Most of GVs have a globular form (Fig. 2A). The size of such GV was estimated by measuring the linear extension of the cross section, *d,* as shown in Fig. 2A. The size of elongated GVs was estimated by measuring the dimension at an angle of 45 degrees with respect to the semiaxes of the cross section (Fig. 2B). Since only a few of the measured GVs had an elongated shape, the choice of the dimension parameter is assumed to have a minor effect on the conclusions.

To assess the adhesion between GVs after the addition of β2-GPI, clearly visible effective angles of contact between the adhered GVs (Y) were measured using Image J software (Fig. 2C).

GVs yield was determined as the proportion of the image surface covered by cross sections of GVs, which was estimated by 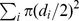, where the summation was performed over all vesicles in the image, and divided by the entire surface of the image *xy*, where *x* and *y* are the width and the height of the image (Fig. 2D).

**Figure 10:**
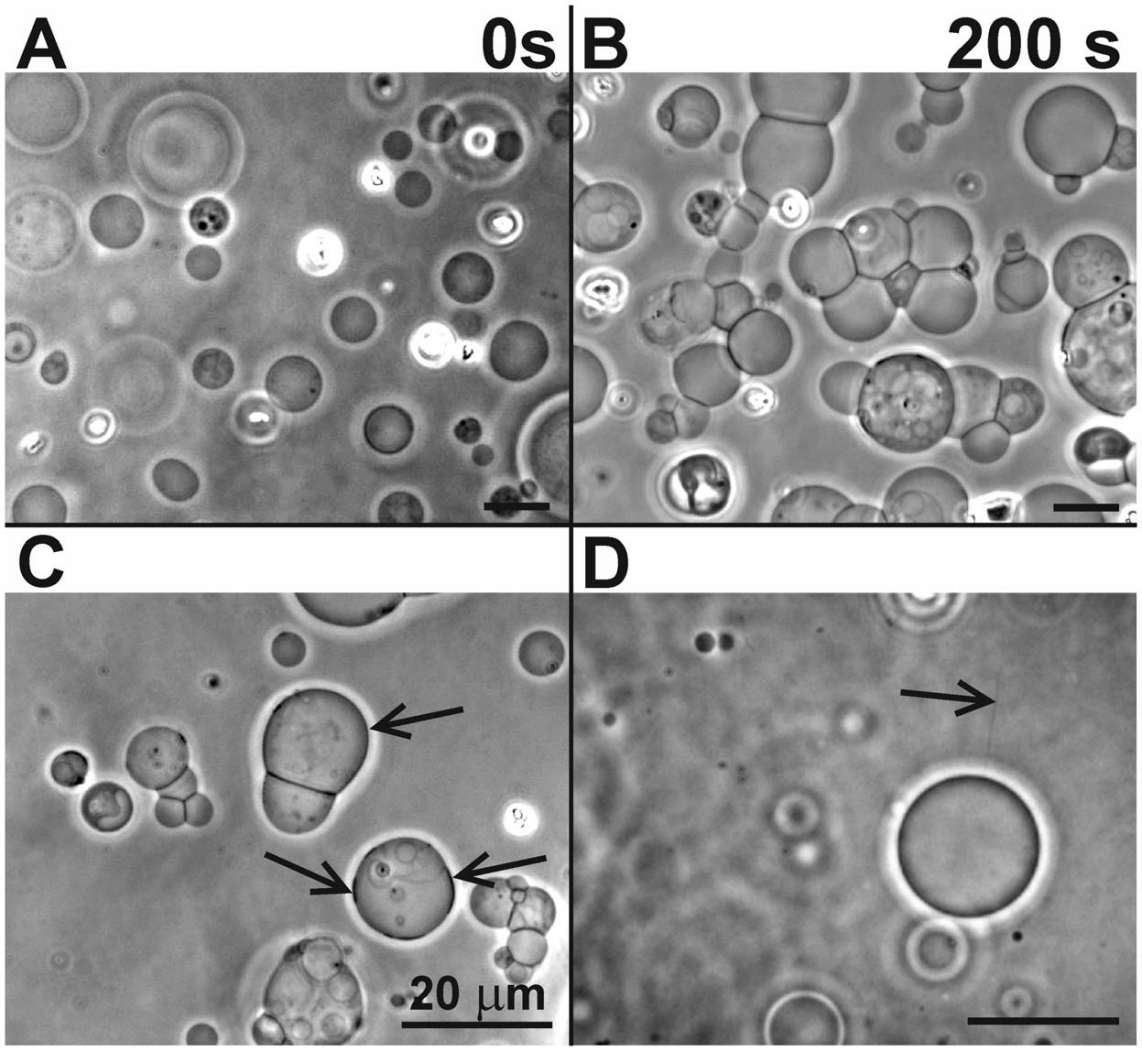
β2-GPI-induced effects on archaebacterial GVs. The effects of β2-GPI on GVs: adhesion (A and B) and lateral segregation of membrane constituents of GVs composed of archaebacterial lipids (S-DGD-5, PGP-Me and PG in proportions 55∶30∶15) (C, marked by arrows). Due to binding of proteins to the membrane, tubular protrusions of GVs (composed of S-DGD-5, aPC, PGP-Me and PG in proportions 25∶30∶30∶15), which are otherwise too thin to be observed by the phase contrast microscope, become visible (D, marked by an arrow). The lengths of all bars are 20 µm.

**Figure 11:**
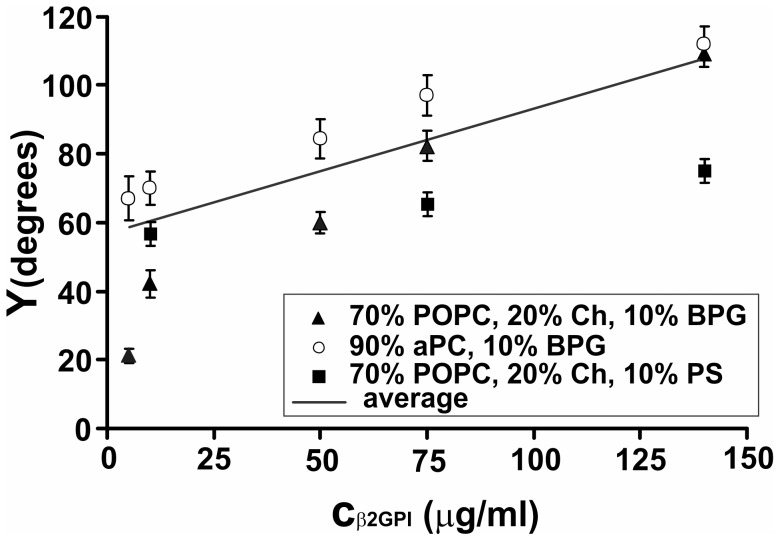
β2-GPI-induced adhesion of non-archaebacterial GVs. Effective angle of contact between the adhering GVs (Y) as a function of β2-GPI concentration.

**Table 1:** Geometrical parameters and adhesion constants for 13 GV pairs adhered due to β2-GPI.

	ϕ_A1_(degrees)	ϕ_A2_(degrees)	av.ϕ_A_(degrees)	rV_1_	rV_2_	av.rV	M	γ_ r_	A_1_(10^−9^m^2^)	A_2_(10^−9^m^2^)	av.A(10^−9^m^2^)	γ(10^−8^J/m^2^)	EAC_1_(degrees)	EAC_2_(degrees)	av.EAC(degrees)
1.	128,8	130,1	128,8	0,96	0,96	0,96	2,12	121	5,18	4,97	5,18	0,42	77,3	80,0	77,3
2.2	133,7	133,3	133,7	0,96	0,98	0,97	2,33	225	2,12	3,43	2,12	1,42	96,6	105,7	96,6
3.	142,2	128,4	142,2	0,95	0,94	0,94	2,43	167	0,98	0,80	0,98	3,27	107,3	98,3	107,3
4.	131,4	129,6	131,4	0,94	0,96	0,95	2,17	135	0,41	0,56	0,41	4,87	98,3	94,7	98,3
5.	140,4	144,9	140,4	0,90	0,89	0,89	2,96	287	0,98	0,84	0,98	5,54	112,1	118,0	112,1
6.	140,8	137,4	140,8	0,92	0,93	0,92	2,68	212	0,94	1,22	0,94	3,44	114,4	107,1	114,4
7.	140,1	135,9	140,1	0,92	0,94	0,93	2,61	225	4,67	7,30	4,67	0,66	96,0	92,1	96,0
8.	134,7	144,2	134,7	0,91	0,94	0,92	2,71	229	3,64	5,11	3,64	0,92	99,7	101,7	99,7
9.	142,2	141,6	142,2	0,88	0,91	0,90	2,90	265	2,60	3,81	2,60	1,45	108,0	104,7	108,0
10.	147,5	147,1	147,5	0,84	0,90	0,87	3,41	385	1,32	2,12	1,32	3,92	126,4	117,7	126,4
11.	138,0	138,9	138,0	0,96	0,95	0,95	2,63	359	5,19	4,12	5,19	1,35	102,5	117,5	102,5
12.	142,6	133,7	142,6	0,94	0,95	0,95	2,62	251	2,14	2,51	2,14	1,89	114,6	103,6	114,6
13.	142,9	138,9	142,9	0,95	0,96	0,95	2,82	471	1,56	1,89	1,56	4,78	103,7	97,1	103,7
av.	138,9	137,2	138,9	0,9	0,9	0,9	2,6	256,3	2,4	3,0	2,4	2,6	104,4	102,9	104,4
SD	5,1	5,9	5,1	0,0	0,0	0,0	0,3	96,0	1,6	2,0	1,6	1,7	11,4	10,6	11,4

ϕ_A1_, ϕ_A2_ - adhesion angles on both sides of adhered GV pair (degrees), av. - average, rV_1_, rV_2_ - volumes of GV relative to volume of untruncated sphere with equal radius, M - tan ((ϕ_A1_+ϕ_A1_)/2), γ_r_ - reduced adhesion constant, A_1_, A_2_ - surface areas of both GVs (10^−9^m^2^), γ -adhesion constant (10^−8^J/m^2^), EAC_1_, EAC_ 2_ - effective angles of contact on both sides of adhered GV pair (degrees), SD - standard deviation.

### Assessment of Mediated Interaction between Membranes

The adhesion constant between a pair of adhered GVs γ was determined according to a recently developed method [39] based on the measurement of adhesion angle *ϕ*_A_ (Fig.3),
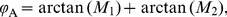
(1)where *M*_1_ = B_1_C_1_/AC_1_ and *M*_2_ = B_2_C_2_/AC_2_, while B_1_C_1_, AC_1_, B_2_C_2_ and AC_2_ are geometrical parameters depicted in Fig.3. To assess the adhesion constant γ, we have used the dependence of the parameter *M*,
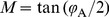
(2)on the reduced adhesion constant
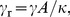
(3)where κ is the membrane bending constant and A is the area of each vesicle [39] which was calculated by assuming mirror symmetry of interacting vesicles while vesicle shapes were determined by minimization of the membrane free energy [39]. Axial symmetry of vesicles was assumed which means that the adhered vesicles have shapes similar to spheres with truncated caps. The parameter *M* depends on the relative volume of adhering vesicles,
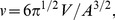
(4)where *V* is the vesicle volume.

The relative volume of the GV is estimated as a volume of the truncated sphere with radius *R* (Fig. 3) and height of the spherical cap *h*, divided by the volume of the entire sphere,
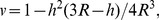
(5)while the area of the vesicle is



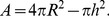
(6)As the adhesion angle was found to show a statistically significant correlation with the effective angle of contact Y [39], we used the effective angle of contact Y to assess the mediated interaction on a population of GVs. To assess the effect of β2-GPI on the adhesion between GVs, we included all clearly visible effective angles of contact between the adhered GVs. We used Image J software (Fig. 2C).

### Statistical Analysis

The populations of GVs were characterized by the average values and standard deviations (SD) of the vesicle dimensions and contact angles calculated with MS Excel (V. 14.0.6112.5000, 32-bit, Microsoft Corporation, Redmond, WA, USA) and OriginPro (V. 8.5.0, OriginLab Corporation, Northampton, MA, USA) software. The statistical significance corresponding to differences between groups of GVs with different lipid compositions (p value) was calculated by the t-test using SPSS software. To determine the connection between variables, linear dependences were assumed and represented by the slope. The statistical significance of the correlations were represented by the Pearson coefficient (r ) and the corresponding probability (p) expressing the statistical significance of the correlation. MS Excel and SPSS (V. 20.0, IBM Corporation, Armonk, NY, USA) software tools were used.

## Results

### Morphology of GVs

We successfully created GVs composed of single species of archaebacterial phospholipids (lipid structures and images in Fig. 4), mixtures of different archaebacterial phospholipids and glycolipids (Fig. 5) and mixtures of archaebacterial and non–archaebacterial lipids in various proportions. In particular, in Fig. 4, GVs constituted of pure archaeal phosphatidylcholine (aPC, from Avanti Polar Lipids) and three (non-commercial) anionic lipids isolated and purified starting from the total lipid extract of an archaeon of the genus *Halorubrum*, are shown; phosphatidylglycerol-phosphate-methylester (PGP-Me) and the sulfoglycolipid (S-DGD-5) are generally present in high proportions in the membrane; while BPG is present in various proportions depending on the experimental conditions [40], [41]. Representative image in Fig. 4A shows numerous GVs obtained from aPC and less numerous vesicles in the preparations of the negatively charged S-DGD-5 and PGP-Me (Fig. 4 B,C). Small crystal-like structures were found in pure PGP-Me GVs (Fig. 4C, marked by arrows). Attempts to create GVs from pure BPG (the archaeal analog of cardiolipin) were unsuccessful; only singular, irregularly-shaped GVs were found (Fig. 4D, top), but many larger (around 20 µm), rounded, crystal-like structures were observed (Fig. 4D, bottom).

Fig. 5 shows GVs composed of low proportion (20%) of anionic archaeal phospholipids and glycolipids and zwitterionic aPC or phosphatidylcholine (POPC), in particular, GVs containing S-DGD-5 (A and B) or PGP-Me (C and D). Also GVs constituted only of negatively charged archaebacterial lipids, present in the same proportions as found in archaea of the genus *Halorubrum* (55% SDGD5∶ 30% PGPMe : 15% PG) were created (Fig. 5E).

After addition of NAO to GVs, we observed flourescence in GVs composed of 60% POPC : 20% cholesterol with either 20% cardiolipin or 20% S-DGD-5, 70% POPC : 20% cholesterol : 10% BPG, 60% POPC : 20% cholesterol : 20% PGP-Me and 80% S-DGD-5∶ 20% BPG. We observed no fluorescence in 80% POPC : 20% cholesterol GVs (data not shown).

Statistical analyses of GVs size and yield are shown in Figs. 6 and 7. The average size of GVs in populations with different lipid compositions (in particular, a different content of archaebacterial phosphatidyl choline (aPC)) are shown in Fig. 6A, B. Pure aPC and POPC formed GVs of average size 18±4 µm and 50±25 µm, respectively. In GVs created of POPC and cholesterol the average size was 43±18 µm, while the addition of archaebacterial phospholipids in general caused a decrease in the average size of GVs (Fig. 6C), i.e., the average size was positively correlated with the weight % of POPC with the slope equal to 0.87 µm per weight %. The correlation was statistically significant (r = 0.625, p<0.0001). Also in GVs created from aPC the average size of GVs decreased (Fig. 6C), i.e., the size was positively correlated with the weight % of aPC, however with smaller slope (0.18 µm per weight %). The correlation was statistically significant (r = 0.16, p<0.0001). The average size of GVs constituted of negatively charged archaebacterial lipids, present in the same proportions as found in archaea of the genus *Halorubrum* (55% SDGD5∶ 30% PGPMe : 15% PG) was 19±9 µm which does not differ from the average size of vesicles composed of aPC (19±5 µm, p = 0.3).

The GV yield (proportion of the area of cross sections of GVs which covers the micrograph frame) is given in Fig. 7. The yield increased with the weight % of aPC (Fig. 7C) with the slope equal to 0.79% coverage per w% aPC, r = 0.52, p = 0.039. Also in GVs composed of mixtures of archaebacterial lipids and non-archaebacterial lipid POPC, the yield increased with the weight % of POPC (Fig. 6C) with the slope 2.29% coverage per w% aPC (r = 0.87, p = 0.16) whereas the yield of 60% POPC : 20% cholesterol : 20% cardiolipin GVs was similar as in the analogue system composed of 80% aPC : 20% BPG (47% versus 38%, respectively), (Fig. 7A,B). In GVs composed of lipids in similar proportions as found in archaea of the genus *Halorubrum*, the yield of GVs was much smaller than in GVs composed of pure aPC (11% versus 99% ) (Fig. 7A).

### DSC Measurement of MLVs

The DSC scan (dependence of the excess specific heat at constant pressure <*C*_p_> on the temperature) of MLVs composed of aPC shows a single relativetly low and wide peak (ΔT full width at half maximum, FWHM = 7.6°C) at T_m_ = 34.2°C with ΔH = 0.66 kcal/mol K (Fig. 8) while the DSC scan of MLVs composed of DPPC shows two narrow peaks pertaining to the main transition at T_m_ = 40.9°C with ΔH = 6.59 kcal/mol K and FWHM = 1.8°C, and the pretransition at T_m_ = 35.5°C with ΔH = 0.68 kcal/mol K and FWHM = 4.3°C (Fig. 8).

### Mediated Interaction Study

We observed a fraxiparine-mediated interaction between archaebacterial lipid-containing GVs (Fig. 9). After addition of 5 µl fraxiparine (diluted in 0.2 M glucose), GVs composed of 75% aPC : 25% BPG adhered to each other in the timescale of minutes. The average contact angle between the vesicles increased with time, reflecting the increase of the area of contact between the vesicles. After the addition of fraxiparine, an increase of membrane permeability to glucose and saccharose was observed in some GVs as a fading of the phase contrast halo (which is the consequence of a different optical density of the GV’s interior (0.2 M sacharose) with respect to its exterior (a mixture of 0.2 M saccharose and 0.2 M glucose)).

β2-GPI - mediated attractive interaction between GVs was observed in a timescale of minutes (Fig. 10A,B) while adhesion to the bottom of the observation chamber took place, similarly as in heparin-induced adhesion [37]. Curvature-induced lateral segregation of membrane constituents (Fig. 10C) and thin tubular structures (Fig. 10D) were also observed. The average effective angle of contact between GVs (Y) increased with increasing β2-GPI concentration (Fig. 11) in all GV systems with different phospholipid compositions. The slope of the median contact angle as a function of β2-GPI concentration was 0.318 degrees per mg/ml of β2-GPI (r = 0.68 and p = 0.005).

We estimated the adhesion constant γ of 13 pairs of GVs which adhered due to the mediating effect of β2-GPI (Tab. 1). For membrane bending constant we used the value in the range of values obtained for different eukaryal lipids (1.75×10^−19^ J) [42]–[47]. The average value of γ was 2.6×10^−8^ J/m^2^ with SD 1.8×10^−8^ J/m^2^ (Tab. 1) while Pearson coefficient of the correlation between γ and the effective contact angle was 0.598 with statistical significance p = 0.018.

## Discussion

Using the electroformation method we created GVs composed of pure archaebacterial lipids and of different mixtures of archaebacterial and non-archaebacterial lipids.

Previous studies considered lipid extracts composed of all lipid components of the membrane, of phospholipids and phosphoglycolipids (especially bipolar tetraether lipids) [9], [15], [16] and also mixtures of tetraether lipids with standard lipids [33]. Recently, it has been shown that liposomes consisting of diether lipids isolated from hyperthermophilic archaea *Aeropyrum pernix* have many physicochemical properties similar to those composed of tetraether lipids [48]. Novelty of our work is that the vesicles are giant and that they have been prepared by mixtures of non commercial individual archaeal lipids isolated and purified in our laboratory, including pure negatively charged diether lipids.

It is of interest to compare archaeal and non-archaeal (standard) lipids, when possible. In general, the archaeal analog of standard lipids (archaeal PC versus standard PC (POPC for example)) have the same headgroups but different chains; other examples are archaeal PG versus standard PG and archaeal BPG versus standard BPG (i.e. cardiolipin). Pure glycolipid GVs or glycolipid rich GVs might be important in clinical applications because of their adjuvant properties [10].

We studied the influence of lipid composition on the size and yield of these GVs.

In general, phosphatidyl choline GVs were the largest and most abundant while addition of other species decreased the size and the yield of GVs both, archaeal and non-archaeal systems. Vesicles constituted of aPC were smaller than vesicles constituted of POPC. As the polar heads of aPC and POPC are identical, the effect can be attributed to the lipid tails. It seems that branched chains increase the curvature. The proportions of the areas of headgroups and chains determine the prefered curvature [49]. Larger area pertaining to headgroups with respect to the area pertaining to the chains implies larger curvature, so it is indicated that branched chains are more compactly packed in the bilayer. Low yield of GVs composed of a lipid mixture similar to that present in natural membranes of microorganisms of *Halorubrum* genus could be a consequence of the absence of salts in the medium, since ions in the medium are important in stabilizing vesicles composed of negatively charged lipids.

BPG was unfavourable for creation of GVs within the given electroformation method. This is in agreement with our attempt to create GVs of cardiolipin within the same electroformation method, which had been unsuccessful previously [50]. We found only singular GVs in the sample obtained from BPG while many crystal-like particles were observed. The question remains whether these singular GVs were actually composed of remnants of other lipids on the electroformation electrodes. According to Israelachvili [49], lipid self-assembly results from the balance of interaction free energy, entropy and molecular geometry which determines local and global shape of a vesicle. The concept of Israelachvili was further elaborated by including orientational ordering of membrane constituents [51], [52]. The flexibility and reorientational mobility of cardiolipin is impaired which favours highly anisotropic membrane curvature and enhances the propensity of cardiolipin to form strongly curved non-lamellar phases [53] (such as inverted hexagonal and cubic phases which can also be present in other lipid systems [54], [55]).

Bagatolli et al. [25] have previously used archaeal tetraether lipids to prepare GVs for the study of configuration of fluorescence probe Laurdan in the membrane composed of the polar lipid fraction E (PLFE) from the thermoacidophilic archaebacteria *Sulfolobus acidocaldarius.* They have observed spherical vesicles while it was previously found that the majority (95%) of GVs created by this method are unilamellar [20]. In our samples, albeit prepared with the same method, there was a considerable proportion of nonspherical or multilamellar vesicles, some of them enclosing obvious internal structures. Also in our samples GVs appear spherical immediately after the formation. However, we performed experiments the next day. Until used, the suspension with vesicles was left for sedimentation in the gravity field to increase the concentration of GVs at the bottom of the tube. As almost all vesicles have attached remnants of the nanotubular network which is formed in electroformation and torn when GVs are rinsed from the observation chamber, with time, the difference between the areas of the outer and the inner membrane layer decreases and attached nanotubes become thicker and shorter. Eventually, they are integrated in the mother vesicle which becomes flaccid and subject to stronger flickering. As the process continues, invaginations appear and are internalized by the mother vesicle to finally yield globular vesicles with numerous internal structures. This process is common in GVs created by electroformation [56]. As many GVs composed of archaebacterial lipids had internal structures, it is indicated that after the formation they are presented with a large pool of membraneous nanostructures attached to GVs. Also, the portion of GVs with obvious internal structures was on the average larger for smaller GVs and for GVs with larger PC content (not shown).

The mixture of lipids in solvent was applied to the electroformation electrodes manually, which may have resulted in an uneven distribution of lipid in different areas. Further, lipid segregation can occur during the electroformation as lipids of a certain species have preference for a given curvature and respond differently to the AC electric field. A nonuniform lateral distribution of lipid species may contribute to the heterogeneity of GVs in the population.

In determining the GV yield, both, the GVs’ size and their number have to be taken into consideration. Also, there are several layers of GVs, while the focus of the microscope is on the cross section revealing a single layer. Another method to assess the GV yield would be the use of flow cytometry for counting GVs [57].

Different lipids in mixtures used for creation of GVs have different (temperature dependent) solubility properties in the organic solvents used for lipid storage. The electroformation method was performed at room temperature for all the lipid mixtures. To attain a greater yield of GVs composed of lipids with a higher gel to liquid crystalline phase transition temperature, different electroformation temperatures should be considered, however, it should be taken into account that polar lipid membranes of archaea are assumed to be in the liquid crystalline phase over a wide temperature range 0–100°C.

GVs were examined by applying the fluorescent marker NAO which was considered to bind selectively to cardiolipin, but was recently shown to also bind to other archaebacterial lipids [58]. Our observations confirm that binding of NAO to lipids is not cardiolipin-specific.

We have used the differential scanning calorimetry to determine the transition temperature of lipid vesicles prepared from pure aPC and from its non-archeal structural analogue DPPC. DSC was performed on MLVs, since we were not able to produce a sufficient amount of GVs to reach the the required lipid concentration for measurement with DSC. However, the curvature of most membranes in MLVs as well as in GVs can be considered very small and therefore both systems can be considered as equivalent in this respect. The DSC scan of MLVs composed of aPC showed one relativetly low and wide (barely recognisable) peak while the DSC scan of MLVs composed from DPPC showed two well defined peaks corresponding to the main phase transitions and the pretransition (Fig. 8). The observed minuteness of the single peak in aPC may contribute to higher yield of aPC GVs comparing to DPPC by electroformation at the room temperature (data not shown).

The attractive interaction between archaebacterial and non-archaebacterial membranes can be mediated by the same molecules as in eukaryotic membranes so the archaeosomes could approach the cell membrane very closely which is a prerequisite for the interaction and uptake of archaeosomes by the cell to take place. We have studied the effect of two relevant molecules (beta 2 glycoprotein I and heparin) and found that they both act similarly in mediating attractive interaction between archaeal and standard membranes. Since mediated interaction mechanisms are non-specific [32] (they derive from the charge distribution in the membrane surface, the shape of mediating molecules and charge distribution within them [59]–[61] and on the preferential orientation of water molecules near the membrane [62]), we assumed and finally have shown that the mediated interaction between the GVs composed of archaebacterial lipids takes place. The mechanism of the interaction is based on the minimization of the collective free energy of the membrane and of the adjacent solution which depends on the orientational ordering of molecules with internally distributed charge (e.g., protein and water molecules) in spatially varying electric field. In archaeal membranes effects similar to the ones observed in non-archaeal membranes were expected since the headgroups which essentially determine the electric field in the vicinity of the membrane are similar in archaeal phospholipids and in non-archaeal phospholipids. As the fusion of vesicles with target cells is a possible mechanism of drug delivery into the cells, it is of relevance to show that blood proteins can promote adhesion or fusion between vesicles constituted of archaebacterial lipids and of mixtures of archaebacterial and standard lipids. This is especially important since it was found that archaeal liposomes do not fuse easily in conditions which are physiological in vertebrates [63]. Archaeal lipids are in some respect different from non-archaeal ones while in other respects they are similar. This ambivalence could be an advantage, as archaeosomes should be more resistant to enzymes and still be able to interact with the host membrane and perform the delivery. Without the latter, high resistance of archaeosomes would be of no benefit.

Adhesion of phospholipid membranes has been a subject of thorough experimental and theoretical research as it represents an essential step for biologically important processes such as endo- and exo-cytosis and fusion of cells [64], [65]. In particular, these processes are important for the efficiency of drug delivery by liposomes. Experimental studies on lamellar membrane stacks by osmotic stress method, microscopy studies and micropipette aspiration (reviewed in [64], [65]) yielded the adhesion constants between 10^−3^ and 10^−4^ J/m^2^, which is much larger than what we have obtained by the interaction mediated by β2-GPI (of the order of 10^−8^ J/m^2^). In contrast, a method which is based on a minimization of the contact area theoretical description of adhesion [66],
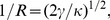
(7)where 1/*R* is the contact curvature, yields values in the same range as our results; in these experiments the contact curvatures obtained from micrographs were of the order of 1/10 µm [67], [68]. It is evident that there is large scattering of data on γ obtained by different methods, mostly due to different experimental techniques used, which implies also different experimental systems. In our opinion, systems of lamellar stacks and interactions with non-membranous materials reveal important physical properties of membranes and adhesion, however, they are further from realistic to be used for drug-delivery systems comparing to systems consisting of populations of adhering GVs observed live by the optical microscopy. Further, studying mediated interaction between GVs includes suggesting models of mediated interaction which are basic for manipulating adhesion. Based on the structure of β2 glycoprotein I and its binding to phospholipid membranes [69]–[71], a bridging interaction was suggested as a mechanism of β2 glycoprotein I-mediated adhesion between GVs [59]. The hydrophobic part of the molecule is inserted into the membrane while the other part with a positively charged region is sticking out and can form a bridge with the membrane of another negatively charged vesicle [59]. In the case of heparin, the relevant mechanism is suggested to be orientational ordering of heparin which bears spatially distributed charge [60]–[62]. This interaction is nonspecific and it does not involve chemical binding of molecules. It could be described as an entropic effect. The membranes do not touch, but are driven very closely together. The distance is smaller than a nanometer, and the area of the contact is maximized, so the effect is interpreted as an adhesion. Both, archaeal and standard lipid GVs satisfy the conditions in which such interaction takes place (they form surfaces between which the mediating molecules are confined) and additionally, both may have charged headgroups, or spatially distributed charge on the headgroups, so they are similar in this respect.

We found statistically significant (p = 0.018) correlation (r = 0.598) between the measured adhesion constant γ and the measured average effective angle of contact Y which justifies the use of the effective angle of contact Y in analysis of the effect of mediating molecules on GV adhesion. Namely, the effective angle of contact Y is considerably less time consuming and also more convenient for assessment of a large number of contacts.

### Conclusion

Archaebacterial lipids and mixtures of archaebacterial and standard lipids readily form GVs. The ability of certain archeal lipids to form GVs in pure form corresponds to the ability of nonarchaeal structural homologue lipids to form GVs. Archaeal, standard and mixed GVs are subject to weak interaction mediated by certain constituents of human blood plasma due to orientational ordering of mediating molecules with internally distributed charge. Mediated adhesion of archaeal vesicles with eukaryotic membranes and an increased resistance of archaeal vesicles to eukaryotic enzymes indicate that archaeal lipid vesicles are potentially superior to standard lipid vesicles as drug carriers.
